# Constrained multivariate association with longitudinal phenotypes

**DOI:** 10.1186/s12919-016-0051-8

**Published:** 2016-10-18

**Authors:** Phillip E. Melton, Juan M. Peralta, Laura Almasy

**Affiliations:** 1 The Curtin/UWA Centre for Genetic Origins of Health and Disease, Faculty of Health Sciences, Curtin University and Faculty of Medicine Dentistry & Health Sciences, The University of Western Australia, Perth, Australia; 2 South Texas Diabetes and Obesity Institute, University of Texas at Brownsville, Brownsville, TX 78520 USA; 3 South Texas Diabetes and Obesity Institute, University of Texas Health Science Center, San Antonio, TX 78229 USA; 4 Department of Biomedical and Health Informatics, The Children’s Hospital of Philadelphia, Philadelphia, PA 19104 USA; 5 Department of Genetics, Perelman School of Medicine, University of Pennsylvania, Philadelphia, PA 19104 USA

## Abstract

**Background:**

The incorporation of longitudinal data into genetic epidemiological studies has the potential to provide valuable information regarding the effect of time on complex disease etiology. Yet, the majority of research focuses on variables collected from a single time point. This aim of this study was to test for main effects on a quantitative trait across time points using a constrained maximum-likelihood measured genotype approach. This method simultaneously accounts for all repeat measurements of a phenotype in families. We applied this method to systolic blood pressure (SBP) measurements from three time points using the Genetic Analysis Workshop 19 (GAW19) whole-genome sequence family simulated data set and 200 simulated replicates. Data consisted of 849 individuals from 20 extended Mexican American pedigrees. Comparisons were made among 3 statistical approaches: (a) constrained, where the effect of a variant or gene region on the mean trait value was constrained to be equal across all measurements; (b) unconstrained, where the variant or gene region effect was estimated separately for each time point; and (c) the average SBP measurement from three time points. These approaches were run for nine genetic variants with known effect sizes (>0.001) for SBP variability and a known gene-centric kernel *(MAP4)*-based test under the GAW19 simulation model across 200 replicates.

**Results:**

When compared to results using two time points, the constrained method utilizing all 3 time points increased power to detect association. Averaging SBP was equally effective when the variant has a large effect on the phenotype, but less powerful for variants with lower effect sizes. However, averaging SBP was far more effective than either the constrained or unconstrained approaches when using a gene-centric kernel-based test.

**Conclusion:**

We determined that this constrained multivariate approach improves genetic signal over the bivariate method. However, this method is still only effective in those variants that explain a moderate to large proportion of the phenotypic variance but is not as effective for gene-centric tests.

## Background

Contemporary analytical approaches to identify individual genetic variants associated with complex disease phenotypes often rely on analyzing phenotypic data from a single time point. However, longitudinal epidemiological studies often collect information on the same quantitative phenotypes from multiple time points [1]. Two recent studies have proposed that joint association analysis of longitudinal phenotypes from repeat measurements increases statistical power to detect genetic variants over univariate methods. Furlotte et al [2] proposed a linear mixed-model approach for association mapping that was able to differentiate genetic, environmental, and residual error components in order to increase power. Fan et al [3] determined that the most successful longitudinal method was a nonparametric penalized linear model. An important consideration is also that effect estimation at each time point allows for the possibility that the genetic association is temporally dependent. Therefore, the incorporation of models that accounts for all time points independently, may have greater statistical power to identify significant effects for complex disease phenotypes when these effects change with time or age. We recently proposed a constrained bivariate approach using whole-genome sequencing data from the Genetic Analysis Workshop 18 (GAW18) that demonstrated an increase in genetic signal for variants that explained a moderate to large amount of the variance of the phenotype and had effects that were stable across time and age [4].

Herein, we extend this method to all available time points in the 200 replicates from the Genetic Analysis Workshop 19 (GAW19) simulated family data. For comparison, we first conducted a univariate approach of the average of systolic blood pressure (SBP) measurements from 3 time points using measured genotype analysis of 9 single nucleotide variants (SNVs). We had previously identified these informative SNVs from our constrained bivariate analysis [4]. We then conducted two multivariate association analyses within the variance-component framework using these same 9 SNVs. Finally, we conducted a gene-centric test under these same conditions for two regions, the *MAP4* region on chromosome 3, and a randomly ascertained equivalent region on chromosome 1, to determine if this was more efficient at identifying genetic association for complex disease.

## Methods

### Data description

The GAW19 family data set contains 849 Mexican American individuals from 20 extended pedigrees from the Type 2 Diabetes Consortium. Each of the 200 simulated data sets includes the following information for each individual for 3 time periods along with gender: age, SBP, diastolic blood pressure, hypertension status, blood pressure medication status, and smoking status [5].

### Univariate association

Maximum likelihood methods were used to determine association for the average SBP measurement across all three time points independently in a measured genotype model available in SOLAR (Sequential Oligogenic Linkage Analysis Routines) [6]. Covariates included age from time point 1, sex, and their interactions as well as smoking status. Variables were carried forward to association models if associated with average SBP at *p* <0.05. This measured genotype model was fitted to the data and compared with the null model of no difference in trait mean by genotype using a likelihood ratio test. Twice the difference in log-likelihoods of these models was distributed as a Chi-square random variable with 1 degree of freedom.

### Multivariate longitudinal association

We also applied maximum likelihood methods in our multivariate longitudinal association analyses. This method investigates the relationship of all highly correlated phenotypes simultaneously. This approach tests the null hypothesis that variance components relating to major gene effects on multiple repeat measurements of a trait are equal to zero. Our proposed method extends standard multivariate variance component methods [7] to investigate the effect of a SNV on the mean trait values of multiple repeat measurements of a phenotype, constraining this effect to be equal for all phenotypic measures, effectively assuming that genetic effects are stable across time or age. The difference between the log-likelihoods of a standard multivariate genetic model, in which a single beta is estimated for the difference in mean trait levels by genotype at each time point versus one in which this beta is constrained to zero, follows a Chi-square distribution with 1 degree of freedom. The mathematical equation has been previously described [4], with the only difference being that we are now constraining the beta coefficients for all available SBP repeat measurements.

For these analyses we used the same covariates from the average univariate analysis along with the same 9 SNVs. We then compared these results to a multivariate model where the effect of the SNV on the mean trait value of each of the three measurements of the phenotype was estimated independently (distributed as a Chi-square distribution with 3 degrees of freedom). Significant results were then added over the 200 GAW19 replicates to determine which method provided the best power to detect association.

### Gene-centric analysis

We applied a gene-centric analysis under a variance component model in SOLAR [8] to two genomic regions: chromosome 1 (encompassing 933 variants from position 47887185 to 48032613) as a null test and *MAP4* (encompassing 933 variants, chromosome 3: 47887577 to 48135350) as a positive test across all 200 simulated replicates. This method applies gene-specific relationship matrices to determine the proportion of the trait’s variance explained by an individual gene as a result of the departure of its localized empirical kinship estimate from the pedigree-derived theoretical kinship estimates. A new variance component parameter (h^2^_geff_) is introduced into the standard variance component model in SOLAR. Significance of each h^2^_geff_ parameter is then obtained from a standard likelihood ratio against the null model and distributed as a ½:½ mixture of a 1-degree-of-freedom Chi-square test and a point mass of 0 [8]. We tested four models for this gene-centric test at a single time point (SBP_1), on the average across all 3 available time points, for all 3 SBP repeat measurements where the effects of the kernel were unconstrained, and finally a model where the kernels were constrained.

## Results and discussion

Table 1 shows the results of three different longitudinal association analyses for nine single nucleotide polymorphisms (SNPs) influencing SBP variation, with effects greater than 0.001, across all 200 GAW19 replicates for *p* values less than 0.001, 5.0E^−5^, and 5.0E^−9^. All analyses identified the *MAP4* variant 3_48040283 as genome-wide significant (*p* value <5.0E^−9^). The *MAP4* SNP, 3_47957996 was significant in 200 of the constrained longitudinal tests and averages tests and in 199 of the unconstrained tests. Additional variants, 1_66075952 from *LEPR* and *MAP4* variants, 3_47956424 and 3_48040284, demonstrated low numbers of genome-wide significant associations for the constrained longitudinal test and only the chromosome 1 variant demonstrated genome-wide significance for the unconstrained method. When compared to our previous GAW18 results using two time points [4] the constrained method utilizing all 3 time points increased power. Averaging SBP across time points appears to be equally effective when the variant has a large effect on the phenotype. However, the constrained method does better with variants with lower effect sizes. The genetic signal for most replicates was reduced in the unconstrained method but maintained in the average method, and this may be a result of the additional degrees of freedom added, making it more difficult to obtain the critical threshold. To ensure that the increased genetic signal from this constrained approach did not come at the expense of an elevated false-positive rate, we chose 20 random SNVs from the simulated model that did not explain any of the variance for SBP. For these 20 null markers, across 200 replicates, *p* values of less than 0.01 were detected 3.5 % of the time for the constrained approach and 0.2 % for the average univariate method. This demonstrates no systematic inflation of *p* values under the null (data not shown).

**Table 1:** Comparisons of association analyses results for 9 functional variants explaining >0.001 % of simulated SBP over 200 GAW19 replicated data sets using measurements from all time points

Variant (%variance SBP^1^)	Multivariate constrained				Multivariate unconstrained			Average all 3 visits	
	0.001	5.0E^−5^	5.0E^−9^	0.001	5.0E^−5^	5.0E^−9^	0.001	5.0E^−5^	5.0E^−9^
3_48040283 (0.0278)	200	200	200	200	200	200	200	200	200
1_66075952 (0.0206)	154	82	2	96	38	1	170	115	8
3_47957996 (0.0149)	200	200	200	200	199	199	200	200	200
3_47956424 (0.0143)	184	150	8	161	100	4	189	165	16
3_48040284 (0.011)	81	29	4	31	9	0	43	11	0
13_28624294 (0.0081)	13	1	0	3	0	0	6	0	0
3_47913455 (0.004)	19	3	0	6	0	0	1	0	0
3_58109162 (0.0027)	50	10	0	15	3	0	2	0	0
19_12541795 (0.0017)	0	0	0	0	0	0	0	0	0

Entries indicate number of replicates meeting *p* value threshold

### Gene-centric tests

As expected, none of the models we tested showed any significant results for the chromosome 1 region. Table 2 shows the results of the gene-centric test for *MAP4*. The average time-point gene-centric test provided higher power than all of the other tests for *MAP4*. This is expected based on the underlying simulation model that has stable genetic effects over time/age and the average is dampening down stochastic effects in these data. It can be assumed that if gene by age interactions were included in the simulated model the average method would do worse. The gene-centric method for the *MAP4* region provided less support than some of the individual SNVs from the same gene. This also may be expected as the gene-centric model provides separate estimates of the proportion of the variance because each time point has the same kernel, which introduces stochastic noise into the data. Consequently, we would expect the constrained multivariate test perform worse than the average. When the constraint is lifted, there is greater statistical noise and the unconstrained test performs the worst. This may be because we incorporated all of the available variants within the gene and did no prioritization based on potential functional or regulatory aspects of the included SNVs.

**Table 2:** Comparison of *MAP4* gene-centric association analysis for regions, with number of times over 200 replicates

	*MAP4*		
	0.001	5.0E^−5^	5.0E^−9^
Single time point (SBP_1)	197	175	55
Average all 3 visits	200	200	135
Multivariate unconstrained	91	32	1
Multivariate constrained	153	90	4

Entries indicate number of replicates meeting *p* value threshold

## Conclusions

In this paper, we present a constrained multivariate approach to increase power to detect association with a variant by constraining the effect of the SNV on the phenotype using a variance-component model for all available measurements of a given phenotype. The model is an extension of our previous work that demonstrated that constraining the beta-coefficient for SNVs across two time points increased genetic signal for variants that had a moderate to large effect on the phenotype over a univariate approach or one where the beta was allowed to fluctuate. The criteria for this constrained method are multiple repeat measurements of the same phenotype or different measurements of the same phenotype (ie, heart rate measured from echocardiograph and electrocardiogram) [9]. Our current results confirm this using additional longitudinal information and indicate improved power with additional time points for variants with moderate to large effect. The model presented in this article demonstrates the efficacy of incorporating longitudinal data into association models for individual variants; however, additional work is necessary for testing this within a gene-centric statistical framework.
